# Emergency Surgery Mortality (ESM) Score to Predict Mortality and Improve Patient Care in Emergency Surgery

**DOI:** 10.1155/2019/6760470

**Published:** 2019-09-23

**Authors:** Sirirat Tribuddharat, Thepakorn Sathitkarnmanee, Pavit Sappayanon

**Affiliations:** ^1^Department of Anesthesiology, Faculty of Medicine, Khon Kaen University, Khon Kaen, Thailand; ^2^Department of Anesthesia, Khon Kaen Hospital, Khon Kaen, Thailand

## Abstract

**Background:**

Emergency surgery has poor outcomes with high mortality. Numerous studies have reported the risk factors for postoperative death in order to stratify risk and improve perioperative care; nevertheless, a predictive model based upon these risk factors is lacking.

**Objective:**

We aimed to identify the risk factors of postoperative mortality and to construct a new model for predicting mortality and improving patient care.

**Methods:**

We included adult patients undergoing emergency surgery at Srinagarind Hospital between January 2012 and December 2014. The patients were randomized: 80% to the Training group for model construction and 20% to the Validation group. Patient data were extracted from medical records and then analyzed using univariate and multivariate logistic regression.

**Results:**

We recruited 758 patients, and the mortality rate was 14.5%. The Training group comprised 596 patients, and the Validation group comprised 162. Based upon a multivariate analysis in the Training group, we constructed a model to predict postoperative mortality—an Emergency Surgery Mortality (ESM) score based on the coefficient of each risk factor from the multivariate analysis. The ESM score comprised 7 risk factors, i.e., coagulopathy, ASA class 5, bicarbonate <15 mEq/L, heart rate >100/min, systolic blood pressure <90 mmHg, renal comorbidity, and general surgery, for a total score of 11. An ESM score ≥4 was predictive of postoperative mortality with an AUC of 0.83. The respective sensitivity, specificity, positive likelihood ratio, negative likelihood ratio, positive predictive value, negative predictive value, and accuracy for an ESM score ≥4 predictive of postoperative mortality was 70.2%, 94.9%, 13.8, 0.3, 69.4%, 95.1%, and 91.4%. The performance of the ESM score in the Validation group was comparable.

**Conclusions:**

An ESM score comprises 7 risk factors for a total score of 11. An ESM score ≥4 is predictive of postoperative mortality with a high AUC (0.83), sensitivity (70.2%), and specificity (94.9%). Four risk factors are preoperatively manageable for decreasing the probability of postoperative mortality and improving quality of patient care.

## 1. Introduction

The mortality rate for patients undergoing noncardiac surgery in Europe—based on the European Surgical Outcomes Study—is 4% with crude mortality rates varying widely between countries (ranging from 1.2% to 21.5%) [1]. By comparison, emergency surgery has poorer outcomes and a higher mortality rate with recent studies reporting the 30-day mortality to be between 14 and 15% [2, 3]. Several studies have reported the risk factors for postoperative death in aid of risk stratification and perioperative care improvement [2–10]. A predictive model constructed from these risk factors would further these aims. The objectives of the current study were (a) to identify the risk factors for postoperative mortality in emergency surgery and (b) to construct a new model to predict mortality and improve patient care.

## 2. Materials and Methods

This was a retrospective and analytical study. The study was approved by the Khon Kaen University Ethics Committee in Human Research (HE581131). Since patient identification was concealed, informed consent was waived. The data extracting sheet did not contain the name or hospital number of the patient, so a unique study number was generated.

We aimed to include approximately 50 risk factors. According to Tabachnick and Fidell [11], to avoid overfitting, the required sample size (*n*) for a logistic regression of a full model should be 50 + 10(*k*). In our study, this meant a total of 580 patients were required based on 50 relevant clinical risk factors (*k*) and a dropout margin of 5%. All patients aged 18 or over undergoing emergency surgery at Srinagarind Hospital between January 2012 and December 2014 were thus included. Patients undergoing cesarean section were excluded. Patient data from medical records were extracted and analyzed.

### 2.1. Statistical Analyses

In order to assess the fit of the developed model, the total recruited sample was randomly divided into 2 groups: 80% for the Training group to construct the predictive model and 20% for the Validation group to validate the model. Each risk factor in the Training group was assessed for the area under the receiver operating characteristic curve (AUC) and the crude odds ratio using a univariate logistic regression analysis. The risk factors with a *P* value ≤0.1 were included in the multivariate logistic regression analyses to identify relevant risk factors. The coefficients of each risk factor derived from the multivariate analysis were used to construct a predictive model. The discriminating ability of the model was assessed by evaluating the AUC. We determined the sensitivity, specificity, positive likelihood ratio, negative likelihood ratio, positive predictive value, negative predictive value, and accuracy of the model. The model was then validated in the Validation group by calculating the AUC and the corresponding performance. Statistical analyses were performed using SPSS version 16.0 (SPSS Inc, Chicago, IL, USA).

## 3. Results

We recruited 758 patients (110 nonsurvivors and 648 survivors). The mortality rate was 14.5%. The Training group comprised 596 patients which exceeded the required calculated sample size, and the Validation group comprised 162. The demographic and clinical data of both groups are presented in Table 1. There were statistically, but not clinically, significant differences in sex and age between both groups.

### 3.1. Model Construction

The demographic and clinical data of nonsurvivors and survivors in the Training group are presented in Table 2. The nonsurvivors were younger and had a higher American Society of Anesthesiologists (ASA) classification, higher preoperative intubation, higher heart rate, lower blood pressure, and lower oxygen saturation. As for comorbidities, the nonsurvivors had more infections and bleeding, higher respiratory rate, renal and coagulation derangement, more arrhythmia, and greater electrolyte imbalance. Non-survivors also had (a) a higher rate of perioperative cardiopulmonary resuscitation (CPR), lactate level, prothrombin time (PT), and international normalized ratio (INR); (b) a lower platelet count, serum bicarbonate, base excess and urine output; and (c) more blood loss leading to more crystalloid, colloid, and blood transfusions.

**Figure 1:**
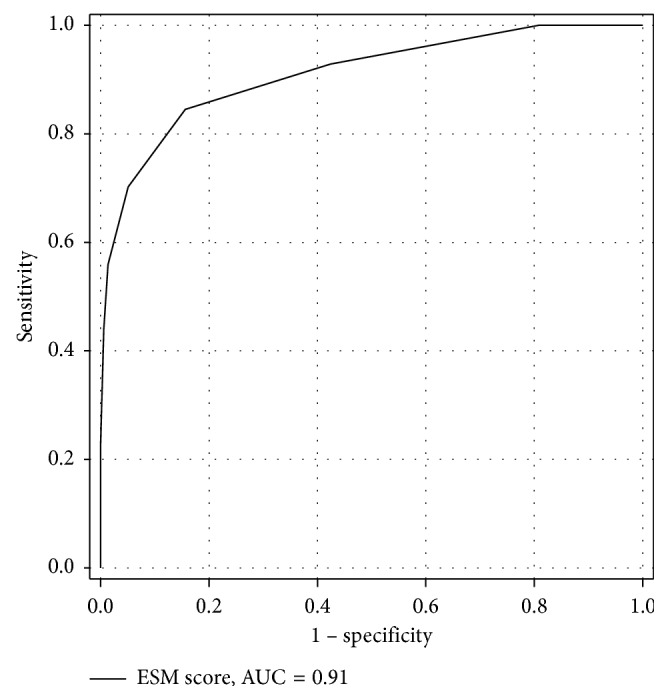
AUC of ESM score to predict mortality in the Training group. ESM: Emergency surgery mortality; AUC: area under the receiver operating characteristic curve.

**Figure 2:**
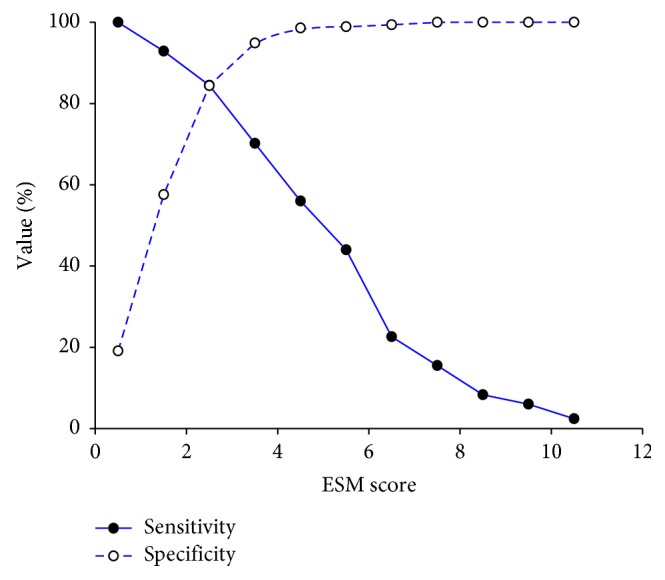
Cutoff point of ESM score to predict postoperative mortality. ESM: emergency surgery mortality.

The AUC, crude odds ratio, and *P* value of relevant risk factors according to the univariate analysis are presented in Table 3. After including all relevant risk factors for multivariate analysis, 7 were identified. We constructed a model—the Emergency Surgery Mortality (ESM) score—to predict postoperative mortality based on the coefficient of each risk factor from the multivariate analysis (Table 4). The ESM score comprised 7 risk factors for a total score of 11. The AUC of ESM score to predict mortality was 0.91 (Figure 1). We identified the cutoff point of the ESM score by plotting a sensitivity and specificity graph (Figure 2). An ESM score ≥4 was predictive of postoperative mortality with an AUC of 0.83.  Table 5 presents the related AUC, sensitivity, specificity, positive likelihood ratio, negative likelihood ratio, mortality prevalence, positive predictive value, negative predictive value, and accuracy at an ESM score ≥4.

### 3.2. Model Validation

**Figure 3:**
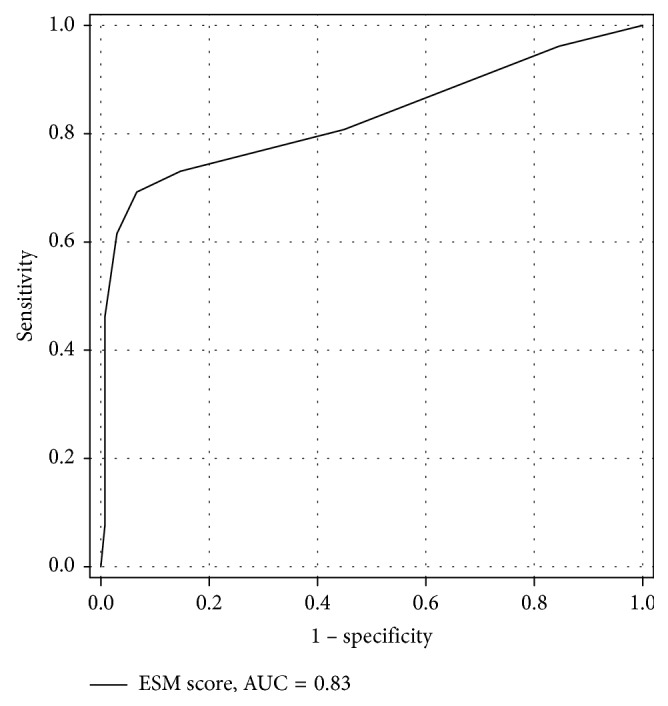
AUC of ESM score to predict mortality in the Validation group. ESM: emergency surgery mortality; AUC: area under the receiver operating characteristic curve.

The ESM score was then applied to the Validation group to assess its performance. The AUC of the ESM score to predict mortality in the Validation group was 0.83 (Figure 3). The discriminative ability of the ESM score to predict mortality in the Validation group is presented in Table 5. The ESM scores in the Validation and Training group are comparable.

## 4. Discussion

The mortality rate of emergency surgery in our study is comparable with that reported in other studies [3–5, 12, 13]. The reported risk factors for postoperative mortality were shock, deteriorated level of consciousness, ischemic heart disease, time from onset of symptoms to hospital admission, a history of cardiac failure, ASA classification, development of in-hospital complications, and preoperative hypotension [4–8]. To our knowledge, no predictive model has been constructed from these risk factors.

The suggested scoring systems for predicting mortality include the POSSUM scoring system and the American College of Surgeons National Surgical Quality Improvement Programme Universal Surgical Risk Calculator [5, 10]. These models are used for clinical triage, decision-making, and quality assessment. Recently, there was a novel Emergency Surgery Acuity Score (ESAS), later called the Emergency Surgery Score (ESS), developed using a multivariate analysis of 18,439 and then validated in 19,552 emergency surgery cases for predicting perioperative mortality in emergency surgery patients [14]. The ESS, having an AUC of 0.86, comprises 22 independent predictors of mortality with a total score range of 0 to 29. The probability of 30-day death gradually increased from 0% to 36% and then 100% at a score of 0, 11, and 22, respectively. This score was later validated in 26,410 cases of emergent laparotomy patients. It was concluded that it could accurately predict mortality in all types of emergent laparotomy patients [15]. The purposes of the ESS score are, however, for preoperative patient counseling and identification of patients needing close postoperative monitoring.

Like the ESS score, the ESM score was constructed from all cases of emergency surgery in our hospital without exclusion of any low-risk surgery so that it could be inferred to all emergency surgery patients in other hospitals. The ESM score has different objectives. We aimed to include as many preoperative factors as possible in order to identify risk factors that can be managed preoperatively to reduce postoperative mortality. We constructed a predictive model from these relevant risk factors in order to (a) assess the prognosis of the patient and (b) improve the quality of patient management.

The ESM score comprises 7 risk factors, i.e., coagulopathy, ASA class 5, bicarbonate <15 mEq/L, heart rate >100/min, systolic blood pressure <90 mmHg, renal comorbidity, and general surgery. The model has a total score of 11 with a cutoff point of ≥4 to predict mortality with high AUC, sensitivity, specificity, and accuracy. From the 7 identified risk factors, 4 are preoperatively manageable (i.e., coagulopathy, bicarbonate <15 mEq/L, heart rate >100/min, and systolic blood pressure <90 mmHg). Coagulopathy is the most important risk factor leading to postoperative mortality with a score of 3 which is near the cutoff point of the model. If an effort is made to identify this factor and promptly correct it preoperatively, the total score of 11 can be reduced by 3, thus reducing the probability of death postoperatively. The other 3 factors (i.e., bicarbonate <15 mEq/L, heart rate >100/min, and systolic blood pressure <90 mmHg) are proxies for inadequate tissue perfusion. A strategy to promptly resuscitate the derangement of the cardiovascular system with an early goal-directed therapy protocol [16, 17] preoperatively can reduce the score by another 1 to 4, hence decreasing both morbidity and mortality.

The ESM score derived from the Training group fits well with the Validation group, underscoring the validity of this score without overfitting. The high AUC of the ESM score (0.91 and 0.83), when applied in both Training and Validation groups, indicates the high predictability of the score. The very high specificity, with high precision of the ESM score, in both the Training and Validation groups (i.e., 94.9% (95% CI 92.7–96.7) and 93.4% (95% CI 87.8–96.9)) indicates the usefulness of this model to predict the patient with ESM score <4 to survive postoperatively with very high accuracy. Thus, if there is prompt, preoperative management of coagulopathy and hypoperfusion—thereby reducing the ESM score to below 4, with the pretest probability to survive of 85.5% (mortality rate 14.5%) and a positive likelihood ratio of 13.8—according to Bayes' theorem, the chances of postoperative survival improves from 85.5% to 98.8%.

The ESM score is simple and can be calculated at bedside. To improve outcomes, the scoring can be done either in the emergency or surgical ward, thereby assessing and resuscitating the patient before they are being transferred to the operating theatre.

## 5. Limitations

Since this was a retrospective study, some of the data may be inaccurate and some relevant risk factors may have been overlooked. This model was constructed from patient data at a single-center university hospital, so inferential applications to other contexts would need further validation.

## 6. Conclusions

The ESM score comprises 7 risk factors (i.e., coagulopathy, ASA class 5, bicarbonate <15 mEq/L, heart rate >100/min, systolic blood pressure <90 mmHg, renal comorbidity, and general surgery). The total score is 11 such that an ESM score ≥4 is predictive of postoperative mortality with a high AUC (0.83) and a respective sensitivity and specificity of 70.2% and 94.9%. Four risk factors could be preoperatively managed so as to decrease the probability of postoperative mortality and improve quality of patient care.

## Figures and Tables

**Table 1:** Demographic data for the Training and Validation groups (*n* = 758).

Parameter	Training group (*n* = 596)	Validation group (*n* = 162)	*P* value
Dead	84 (14.1)	26 (16.0)	0.531
Sex (male)	360 (60.4)	114 (70.4)	0.022
Age (yr)	58.8 ± 8.7	54.3 ± 16.9	0.003
Weight (kg)	60.3 ± 29.4	60.0 ± 12.9	0.914
Height (m)	1.6 ± 0.1	1.6 ± 0.1	0.026
BMI (kg/m^2^)	23.4 ± 10.7	22.9 ± 4.1	0.547
ASA classification			0.223
2E	23 (3.9)	5 (3.1)	
3E	69 (11.6)	28 (17.3)	
4E	446 (74.8)	111 (68.5)	
5E	58 (9.7)	18 (11.1)	

Data are presented as mean ± SD or number (%); BMI: body surface area; ASA: American Society of Anesthesiologists.

**Table 2:** Patient demographic and clinical data of the Training group (*n* = 596).

Parameter	Nonsurvivors (*n* = 84)	Survivors (*n* = 512)	*P* value
General data			
Age (yr)	52.1 ± 20.3	59.9 ± 18.2	<0.001
Sex			
Male	54 (64.3)	306 (59.8)	0.471
Body weight (kg)	62.9 ± 12.5	59.8 ± 1.2	0.108
Height (m)	1.6 ± 0.1	1.6 ± 0.1	0.074
BMI (kg/m^2^)	24.1 ± 4.6	23.3 ± 11.4	0.260
ASA class			<0.001
2E	2 (2.4)	21 (4.1)	
3E	11 (13.1)	58 (11.3)	
4E	37 (44.0)	409 (79.9)	
5E	34 (40.5)	24 (4.7)	
On endotracheal tube	41 (48.8)	131 (25.6)	<0.001
Heart rate (beats/min)	109.4 ± 28.2	94.9 ± 21.8	<0.001
Systolic blood pressure (mmHg)	103.4 ± 33.9	125.2 ± 24.5	<0.001
Diastolic blood pressure (mmHg)	59.4 ± 19.1	71.4 ± 15.0	<0.001
Temperature (°C)	37.2 ± 0.7	38.2 ± 11.5	0.459
Oxygen saturation (%)	93.2 ± 10.7	98.3 ± 4.8	<0.001
Smoking	11 (13.1)	56 (10.9)	0.575
Previous anesthesia	34 (40.5)	235 (45.9)	0.629
Duration of anesthesia (min)	123.0 ± 82.3	123.2 ± 78.5	0.974
Type of surgery			0.440
General	42 (50)	220 (43.0)	
Cardiovascular and thoracic	5 (6.0)	51 (9.9)	
Neurologic	14 (16.6)	124 (24.2)	
Plastic	3 (3.6)	16 (3.1)	
Urologic	5 (6.0)	28 (5.5)	
Orthopedic	6 (7.1)	20 (3.9)	
Otorhinolaryngologic	4 (4.7)	25 (4.9)	
Gynecologic	3 (3.6)	19 (3.7)	
Medical	2 (2.4)	9 (1.8)	
Comorbidity			
Infection	23 (27.3)	96 (18.7)	0.037
Bleeding	27 (32.1)	51 (10.0)	<0.001
Hypertension with cardiovascular sequelae	3 (3.6)	28 (5.5)	0.405
Cardiovascular system	8 (9.5)	74 (14.5)	0.220
Respiratory system	21 (25.0)	85 (16.6)	0.046
Central nervous system	27 (32.1)	134 (26.2)	0.157
Renal	50 (59.5)	218 (42.6)	<0.001
Liver	17 (20.2)	90 (17.6)	0.543
Coagulopathy	27 (32.1)	5 (1.0)	<0.001
Diabetes mellitus	24 (28.6)	148 (28.9)	0.885
Arrhythmia	26 (30.9)	102 (19.9)	0.009
Electrolyte imbalance	66 (78.6)	280 (54.7)	<0.001
Postcardiac arrest	29 (34.5)	21 (4.1)	<0.001
CPR preoperative	24 (28.6)	3 (0.6)	<0.001
CPR intraoperative	31 (36.9)	2 (0.4)	<0.001
Operating room set >2 h	13 (15.5)	141 (27.5)	0.007

Investigation			
Hemoglobin (g/dL)	9.4 ± 2.9	9.6 ± 2.5	0.669
Hematocrit (%)	28.1 ± 8.4	29.1 ± 7.2	0.279
White blood count (×10^3^/*μ*L)	16.40 ± 12.26	13.83 ± 10.70	0.074
Platelet count (×10^3^/*μ*L)	158.9 ± 84.1	224.1 ± 127.5	<0.001
Blood urea nitrogen (mg/dL)	30.1 ± 23.7	32.6 ± 27.4	0.451
Creatinine (mg/dL)	2.2 ± 1.8	2.2 ± 2.6	0.713
Blood sugar (mg/dL)	167.5 ± 86.1	166.1 ± 78.5	0.917
Sodium (mEq/L)	138.6 ± 8.9	135.7 ± 5.7	0.005
Potassium (mEq/L)	4.0 ± 0.8	3.8 ± 0.7	0.161
Bicarbonate (mEq/L)	16.8 ± 6.5	21.5 ± 4.5	<0.001
Base excess (mEq/L)	−(12.4 ± 6.9)	−(4.4 ± 8.2)	<.0001
Chloride (mEq/L)	100.9 ± 9.8	99.1 ± 6.3	0.111
Lactate (mmol/L)	8.3 ± 4.9	3.9 ± 3.6	<0.001
Albumin (g/dL)	2.5 ± 0.7	2.9 ± 2.4	0.258
Bilirubin (mg/dL)	5.3 ± 7.2	2.4 ± 3.1	0.103
PT (sec)	17.1 ± 6.3	14.0 ± 4.4	0.001
INR	1.7 ± 0.6	1.3 ± 0.4	<0.001
PTT (sec)	42.6 ± 21.8	36.2 ± 15.8	0.030
ALT (unit/L)	66.6 ± 107.5	78.3 ± 155.2	0.751
AST (unit/L)	92.0 ± 79.7	88.8 ± 188.3	0.943
Alkaline phosphatase (IU/L)	130.2 ± 124.2	201.5 ± 180.4	0.098
CXR with significant finding	6 (7.1)	89 (17.4)	0.007
EKG with significant finding	19 (22.6)	96 (18.8)	0.339
Arterial line	50 (59.5)	180 (35.2)	<0.001
CVP (cmH_2_O)	9.3 ± 8.2	12.0 ± 6.4	0.018

Drug			
Inhalation (*n*) (desflurane/sevoflurane)	20 (23.8)/64 (76.2)	102 (19.9)/410 (80.1)	0.413
Narcotic (*n*) (morphine/fentanyl)	1 (1.2)/83 (98.8)	6 (1.2)/506 (98.8)	0.988
Induction (*n*) (propofol/etomidate)	59 (70.2)/25 (29.8)	385 (75.2)/127 (24.8)	0.334
Midazolam	36 (42.9)	103 (20.1)	<0.001
Nitrous oxide	0	13 (2.5)	0.159
Neostigmine	0	13 (2.5)	0.158
Dopamine or dobutamine	40 (47.6)	58 (11.3)	<0.001
Adrenaline	53 (63.1)	16 (3.1)	<0.001
Noradrenaline	47 (56.0)	113 (22.1)	<0.001

Fluid intake			
Crystalloid (mL)	1,048.4 ± 870.1	661.4 ± 710.4	<0.001
Colloid (mL)	583.3 ± 927.9	159.4 ± 327.4	<0.001
PRC (unit)	3.6 ± 6.0	0.6 ± 1.3	<0.001
FFP (unit)	2.5 ± 4.3	0.4 ± 0.9	<0.001
Platelet (unit)	2.6 ± 3.9	0.8 ± 2.2	<0.001
Cryoprecipitate (unit)	0.5 ± 2.0	0.1 ± 1.0	0.033
Urine output (mL)	51.4 ± 117.1	156.7 ± 264.3	<0.001
Blood loss (mL)	1,612.2 ± 3,165.4	261.5 ± 675.3	<0.001

Data are presented as mean ± SD or number (%); ASA: American Society of Anesthesiologists; CPR: cardiopulmonary resuscitation; PT: prothrombin time; INR: international normalized ratio; PTT: partial thromboplastin time; ALT: alanine aminotransferase; AST: aspartate aminotransferase; CXR: chest X-ray; EKG: electrocardiogram; CVP: central venous pressure; PRC: pack red cell; FFP: fresh frozen plasma.

**Table 3:** Relevant risk factors according to univariate analysis.

Parameter	AUC	Crude odds ratio	*P* value
ASA class 5	0.69	14.5	<0.001
On endotracheal tube	0.62	2.8	<0.001
Heart rate >100 beats/min	0.69	5.4	<0.001
Systolic blood pressure <90 mmHg	0.70	16.1	<0.001
Type of surgery			
General surgery	0.57	1.8	0.010
Comorbidity			
Renal	0.60	2.2	<0.001
Coagulopathy	0.65	71.9	<0.001
Arrhythmia	0.57	2.1	0.004
Electrolyte imbalance	0.62	3.2	<0.001
Postcardiac arrest	0.65	10.7	<0.001
CPR preoperative	0.63	45.3	<0.001
CPR intraoperative	0.681	225.4	<0.001
Operating room set >2 h	0.45	0.56	0.008
Investigation			
White blood count >12 × 10^3^/*μ*L	0.58	2.0	0.014
Platelet count <8 × 10^3^/*μ*L	0.55	2.7	0.005
Creatinine >2.3 mg/dL	0.59	2.1	0.002
Bicarbonate <15 mEq/L	0.70	12.4	<0.001
Base excess >−7 mEq/L	0.71	6.3	<0.001
Lactate >5 mmol/L	0.75	9.1	<0.001
Albumin <2.5 g/dL	0.59	2.1	0.032
Bilirubin >1 mg/dL	0.65	4.0	0.019
PT >14 sec	0.63	3.0	<0.001
INR >1.5	0.66	4.4	<0.001
PTT >33 sec	0.60	2.2	0.005
AST >55 unit/L	0.64	3.3	0.017
Drug			
Midazolam	0.62	3.0	<0.001
Dopamine/dobutamine	0.69	7.5	<0.001
Adrenaline	0.79	42.4	<0.001
Noradrenaline	0.66	4.3	<0.001
Fluid intake			
Crystalloid >1,000 mL	0.64	3.6	<0.001
Colloid >500 mL	0.62	3.1	<0.001
PRC >2 units	0.65	4.5	<0.001
FFP >2 units	0.65	4.5	<0.001
Platelets >4 units	0.60	3.6	<0.001

AUC: area under the receiver operating characteristic curve; ASA: American Society of Anesthesiologists; CPR: cardiopulmonary resuscitation; PT: prothrombin time; INR: international normalized ratio; PTT: partial thromboplastin time; AST: aspartate aminotransferase; PRC: pack red cell; FFP: fresh frozen plasma.

**Table 4:** Results of multivariate analysis and the emergency surgery mortality (ESM) score.

	Coefficient	*P* value	Adjusted odds ratio	ESM score
Coagulopathy	3.742	<0.001	42.2	3
ASA class 5	2.174	<0.001	8.8	2
Bicarbonate <15 mEq/L	2.043	<0.001	7.7	2
Heart rate >100/min	1.639	<0.001	5.2	1
Systolic blood pressure <90 mmHg	1.514	<0.001	4.5	1
Renal comorbidity	1.341	<0.001	3.8	1
General surgery	0.922	0.008	2.5	1
Total score				**11**

ESM: Emergency surgery mortality; ASA: American Society of Anesthesiologists.

**Table 5:** Performance of ESM score ≥4 to predict postoperative mortality in Training and Validation groups.

Parameter	Training group (*n* = 596)		Validation group (*n* = 162)	
	Value	95% CI	Value	95% CI
AUC	0.83	0.77–0.89	0.81	0.70–0.92
Sensitivity (%)	70.2	59.3–79.7	69.2	48.2–85.7
Specificity (%)	94.9	92.7–96.7	93.4	87.8–96.9
Positive likelihood ratio	13.8	9.3–20.6	10.5	5.3–20.7
Negative likelihood ratio	0.3	0.2–0.4	0.3	0.2–0.6
Mortality prevalence (%)	14.1	11.4–17.2	16.1	10.8–22.6
Positive predictive value (%)	69.4	60.4–77.2	66.7	50.3–79.8
Negative predictive value (%)	95.1	93.3–96.4	94.1	89.9–96.6
Accuracy (%)	91.4	88.9–93.6	89.5	83.7–93.8

ESM: Emergency surgery mortality; AUC: area under the receiver operating characteristic curve.

## Data Availability

The data used to support the findings of this study are available from the corresponding author upon request.
